# The Interaction Between Vasculogenic Mimicry and the Immune System: Mechanistic Insights and Dual Exploration in Cancer Therapy

**DOI:** 10.1111/cpr.13814

**Published:** 2025-01-26

**Authors:** Shutong Liu, Mei Kang, Yuqing Ren, Yuyuan Zhang, Yuhao Ba, Jinhai Deng, Peng Luo, Quan Cheng, Hui Xu, Siyuan Weng, Anning Zuo, Xinwei Han, Zaoqu Liu, Teng Pan, Li Gao

**Affiliations:** ^1^ Department of Interventional Radiology The First Affiliated Hospital of Zhengzhou University Zhengzhou China; ^2^ Medical School of Zhengzhou University Zhengzhou China; ^3^ Department of Respiratory and Critical Care Medicine The First Affiliated Hospital of Zhengzhou University Zhengzhou China; ^4^ Richard Dimbleby Department of Cancer Research Comprehensive Cancer Centre, Kings College London London UK; ^5^ The Department of Oncology Zhujiang Hospital, Southern Medical University Guangzhou China; ^6^ Department of Neurosurgery Xiangya Hospital, Central South University Changsha China; ^7^ Interventional Institute of Zhengzhou University Zhengzhou China; ^8^ Interventional Treatment and Clinical Research Center of Henan Province Zhengzhou China; ^9^ Institute of Basic Medical Sciences, Chinese Academy of Medical Sciences and Peking Union Medical College Beijing China; ^10^ Longgang District Maternity & Child Healthcare Hospital of Shenzhen City (Longgang Maternity and Child Institute of Shantou University Medical College) Shenzhen China; ^11^ Department of Nursing, Sichuan Clinical Research Center for Cancer Sichuan Cancer Hospital & Institute, Sichuan Cancer Center, University of Electronic Science and Technology of China Chengdu China

**Keywords:** angiogenesis, immune system, tumour, vasculogenic mimicry

## Abstract

Vasculogenic mimicry (VM) represents a novel form of angiogenesis discovered in numerous malignant tumours in recent years. Unlike traditional angiogenesis, VM facilitates tumour blood supply independently of endothelial cells by enabling tumour cells to form functional vascular networks. This phenomenon, where tumour cells replace endothelial cells to form tubular structures, plays a pivotal role in tumour growth and metastasis. Tumour progression is influenced by a variety of factors, including immune components. The immune system serves as a critical defence mechanism by identifying and eliminating abnormal entities, such as tumour cells. This inevitably reminds us of the intricate connection between the immune system and VM. Indeed, in recent years, some studies have shown that immune responses and related immune cells play different regulatory roles in the formation of VM. Therefore, this review provides a comprehensive discussion on the mechanisms underlying VM formation, its interplay with the immune system, and the potential of leveraging immunotherapy to target VM.

## Graphical abstract

VM is a novel form of angiogenesis discovered in many malignant tumours in recent years. And the well‐known immune response and related immune cells play different regulatory roles in the formation of VM.

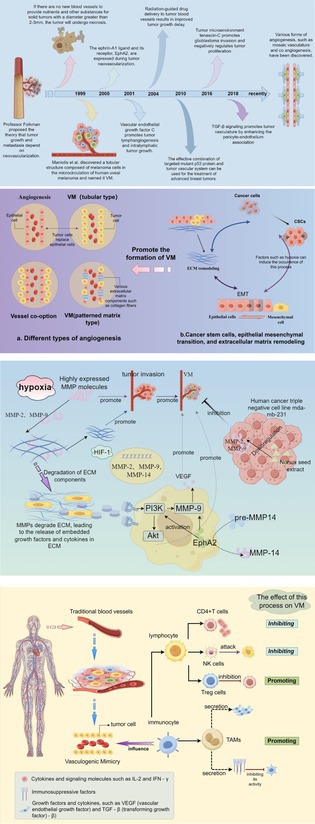

**Abbreviations**
CSCs: cancer stem cells
CT: computed tomography
ECM: extracellular matrix
EMT: epithelial‐mesenchymal transition
EphA2: erythropoietin‐producing hepatocellular A2
FGF: fibroblast growth factor
GBM: glioblastoma
GC: gastric carcinoma
IL‐10: interleukin‐10
MMPs: matrix metalloproteinases
MRI: magnetic resonance imaging
OCT: optical coherence tomography
PAS: periodic acid‐Schiff stain
PECAM1: platelet endothelial cell adhesion molecule‐1
PGF: prostaglandin F
STAT3: signal transducer and activator of transcription 3
TAMs: tumour‐associated macrophages
TGF‐β: transforming growth factor‐β
VDBP: vitamin D binding protein
VE‐cadherin: vascular endothelial cadherin
VEGF: vascular endothelial growth factor
VEGFR‐1: vascular endothelial growth factor receptor 1
VEGFR‐2 (Flk‐1): vascular endothelial growth factor receptor 2
VM: vasculogenic mimicry

## 1 Introduction

Tumour metastasis is the main cause of tumour deterioration and patient death [1]. In 1971, Professor Folkman proposed that tumour growth and metastasis rely on neovascularization, highlighting the necessity of new blood vessels to sustain tumours larger than 2–3 mm in diameter [2, 3]. For many years, this theory formed the basis of anti‐angiogenic therapies, which sought to inhibit the formation of new blood vessels. However, the limited success of anti‐angiogenic treatments revealed the need for a deeper understanding of tumour vascularization. In 1999, Maniotis et al. discovered a novel microcirculatory pattern in human uveal melanoma: a tubular structure composed of melanoma cells, independent of endothelial cells [4]. They found that despite the absence of endothelial cell‐arranged blood vessels in invasive uveal melanoma, the tumour does not necrotize. Therefore, they proposed a novel tumour microcirculation pattern different from the classical tumour angiogenesis pathway, which is named vasculogenic mimicry (VM). The concept of VM not only challenges the traditional theory that endothelial cell‐mediated angiogenesis is the only mechanism for tumour growth and metastasis but also supplements the theory of tumour angiogenesis, providing new theoretical support for researchers to develop new drugs. In subsequent studies, it was found that VM not only exists in uveal melanoma, but also in liver cancer, prostate cancer and ovarian cancer [5]. The presence of VM is closely related to tumour growth, differentiation and invasiveness. Moreover, VM indicates that while current treatments inhibit conventional angiogenesis, they may still support tumour survival and metastasis. The distinctive characteristics of VM make it a promising therapeutic target. However, its clinical application faces obstacles, including drug specificity and resistance mechanisms. Recent studies have started to investigate the potential of combining VM‐targeted therapies with other approaches, such as immune checkpoint inhibitors and anti‐angiogenic therapies, to address these challenges and enhance treatment effectiveness.

On the other hand, the occurrence and development of tumours involve various factors, among which immune factors are one of them [6]. In a healthy human body, the immune system has immune defence, immune monitoring and immune regulation functions [7]. It can help us prevent the invasion of external pathogens and eliminate invading pathogens and other harmful substances. It is also possible to detect and eliminate non‐self components present in the body at any time, such as tumour cells and ageing dead cells. The immune system recognises cancer cells, kills tumour cells and inhibits their growth and proliferation, thereby achieving anti‐tumour goals [8]. The low immune surveillance function greatly increases the probability of tumour occurrence. In addition, some studies in recent years have also shown that the immune system plays an important role in regulating VM [9]. The immune system and vascular mimicry VM are closely related to tumours. Therefore, this article will comprehensively discuss the formation of VM during tumour progression and its interaction with the immune system, and explore the potential of immunotherapy intervention in VM. By comprehensively reviewing and analysing the research progress in related fields, the aim is to deepen the understanding of VM and immunity and provide a scientific basis for future treatment strategies.

## 2 The Characteristics and Formation Mechanism of VM

### 2.1 The Structural Characteristics and Judgement Criteria of VM

#### 2.1.1 Characteristics of VM

VM structure is a special tubular structure formed by highly progressive cancer cells through the interaction of self‐deformation and extracellular matrix remodelling [10], which can be divided into two phenotypes: tubular and patterned matrix [11]. The morphology and characteristics of these two phenotypes differ in tumour tissue. The pipeline structure of tubular VM phenotype is usually supported by scaffolds constructed by tumour cells, which can be single pipelines or branched, networked pipeline networks [12]. In contrast, patterned matrix VM typically involves tumour cells forming aggregates or lattice‐like structures rather than tubular arrangements. These patterns may be grid‐shaped, spotted, or circular [13]. This pipeline has similar functions to traditional blood vessels and can be connected to the original host blood vessels. It provides the necessary oxygen and nutrients for the tumour interior by transporting blood, thereby rebuilding the tumour microenvironment and participating in tumour proliferation and metastasis [14]. However, compared to traditional blood vessels in terms of structure, their pipelines are not composed of endothelial cells, but are lined with tumour cells, and the basement membrane outside the channels is also positive for PAS staining [12]. In addition, the difference between VM and traditional tumour blood vessels lies in the fact that there is a layer of PAS‐positive substance with varying thickness in the VM pipeline that separates tumour cells from the blood flow, and there is rarely any leakage of red blood cells and the formation of micro thrombosis. Rarely do red blood cell leakage and microthrombus formation occur, leading to a decrease in vascular permeability and potentially reducing the efficiency of metabolite and waste clearance [15]. The blood in normal blood vessels will directly come into contact with endothelial cells, and some tumours may also have larger endothelial cell windows in their internal blood vessels, often with red blood cells leaking out [16]. Compared with traditional blood vessels, tumour cells involved in VM exhibit increased plasticity. While endothelial cells in traditional angiogenesis are relatively stable, tumour cells participating in VM formation show remarkable plasticity. They can undergo processes such as EMT to acquire migratory and invasive properties, enabling the formation of these vessel‐like structures. This plasticity makes VM more adaptive, allowing it to overcome therapeutic interventions aimed at inhibiting traditional angiogenesis. Moreover, VM exhibits greater functional heterogeneity. VM structures vary depending on the tumour type and even within the same tumour. While some VM structures can perform basic vascular functions, such as delivering nutrients and oxygen, others may fail to fully integrate with the host vascular network. This heterogeneity makes VM a complex and unpredictable feature of tumours [17].

**FIGURE 1:**
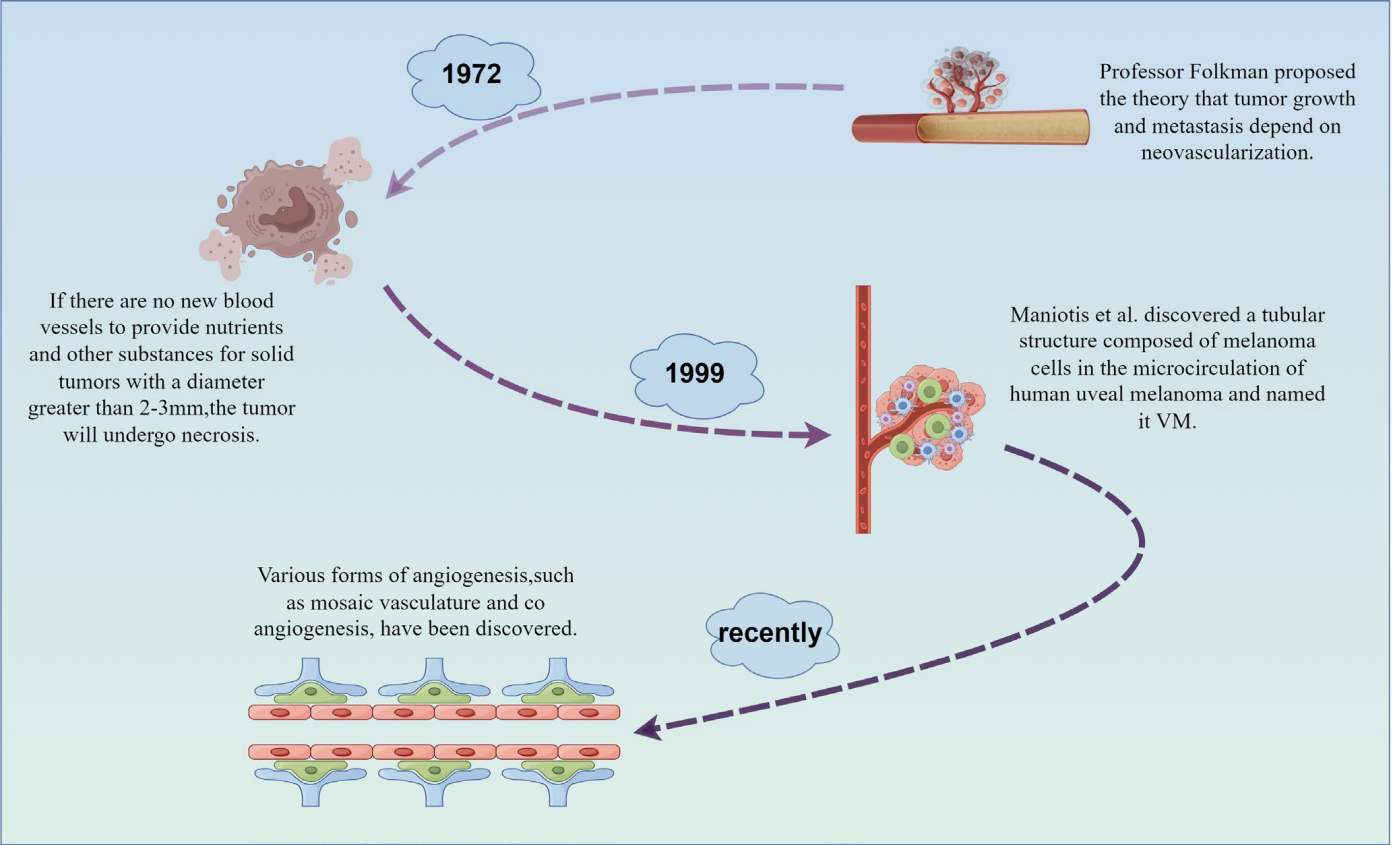
Research progress on tumour vascular morphology. In 1972, Professor Folkman proposed the theory that tumour growth and metastasis depend on neovascularization. With the progress of time, research on the mechanisms related to tumour growth and metastasis has become increasingly in‐depth, and more studies related to angiogenesis have gradually been proposed, such as various forms of tumour angiogenesis such as VM, mosaic blood vessels and vessel co‐option. (By Figdraw.)

VM usually occurs in highly invasive tumours, such as malignant melanoma, breast cancer, lung cancer, liver cancer, etc. Factors in the tumour microenvironment, such as hypoxia, low pH, overexpression of cytokines and growth factors, may also promote the formation of vascular mimicry in tumour cells [18]. In addition, some tumours may develop resistance to traditional treatment methods such as radiotherapy and chemotherapy. In this case, tumour cells may obtain the necessary nutrients and oxygen for survival by forming a vascular mimicry VM, thereby continuing to grow and spread (Figure 1).

Therefore, these structural differences highlight why VM can contribute to tumour progression even in the absence of endothelial‐dependent angiogenesis. In some cases, tumours can thrive and metastasize through VM, particularly when traditional angiogenesis is inhibited, making VM a critical pathway in treatment resistance (Table 1). What's more, as more and more research teams are conducting research on the formation of blood vessels in tumours, in addition to the traditional methods of tumour angiogenesis and VM, mosaic blood vessels and lymph angiogenesis have also been mentioned as a new form of angiogenesis [19, 20].

**TABLE 1:** Further understanding of different types of tumour blood vessels.

Category	Composition	Principle and mechanism	Advantages	Disadvantages	Ref.
VM	Striped tumour cells	Cancer cells acquire endothelial cell‐like characteristics, forming vessel‐like structures within the tumour Cancer cells acquire endothelial cell‐like characteristics, forming vessel‐like structures within the tumour	Provides additional vascular support for the tumour, and enhances tumour cell survival and dissemination within the tumour	This may result in irregular tumour vascular morphology, and increase tumour resistance to treatment drugs	[4, 21]
Angiogenesis	Endothelial cells	New blood vessel formation is induced by tumour cells releasing angiogenic factors (e.g., VEGF), stimulating the proliferation and migration of surrounding endothelial cells to form new vessels	Provides more oxygen and nutrients for the tumour, and accelerates tumour growth and dissemination	May increase tumour vascular density, facilitating easier metastasis.	[2, 22]
Mosaic‐vessel	Endothelial cells and surrounding tumour cells	Mosaic vessels are highly branched and multi‐directional vascular networks composed of multiple small vessel branches and crossovers, forming a unique mesh‐like structure	Increases the complexity of hemodynamics, making it easier for blood to form multiple flow channels within tumour tissue, increasing the possibility of drug delivery	This may lead to unstable blood flow within the tumour, increasing the risk of tumour metastasis	[23]
Vascular Co‐option	Endothelial cells, tumour cells and stromal cells	Utilises existing vessels surrounding the tumour. Due to the interaction between vascular endothelial cells and surrounding tumour cells, these vessels develop resistance to treatment drugs	Provides rapid blood supply, ensuring adequate oxygen and nutrients for tumour cells	This may prevent effective drug delivery to deep tumour regions and increase the complexity and difficulty of treatment as these vessels develop resistance to treatment drugs	[24, 25]

#### 2.1.2 Criteria for Judging VM Structure

Vascular mimicry is a phenomenon in which tumour cells simulate normal blood vessels by forming pipes or tubular structures. Its formation mainly includes the self‐deformation of tumour cells, the remodelling of the extracellular matrix, and the connection with the host blood vessels to provide blood supply to tumour cells and tissues [26]. The main purpose of determining the structure of VM is to distinguish it from real blood vessels. This distinction is crucial for the diagnosis, treatment and prognosis evaluation of tumours. Therefore, determining whether a structure is a VM can be approached from the following aspects.

Firstly, traditional methods for detecting VM involve histological analysis using specific staining techniques. Periodic acid‐Schiff (PAS) staining is commonly employed to identify the presence of VM structures, as the PAS reaction highlights the basement membrane components of vascular structures. VM channels typically exhibit positive PAS staining but lack endothelial cell markers such as PECAM1 and CD34 [27]. Secondly, immunohistochemical techniques have become increasingly important in the detection of VM. Markers such as CD31 (PECAM1), CD34 and von Willebrand factor are commonly used to identify endothelial cells and differentiate them from tumour cells. In VM, these markers are typically absent, while markers such as vimentin (a mesenchymal marker) and certain matrix metalloproteinases (MMPs) that promote extracellular matrix remodelling are often expressed by the tumour cells forming VM channels. This molecular profiling is valuable for confirming VM presence and distinguishing it from traditional angiogenesis. In addition, optical imaging, morphological features and functional verification can also assist in determining VM. Modern biological imaging techniques can image tissue structures through non‐invasive methods, such as fluorescence microscopy, optical coherence tomography (OCT), etc. These methods enable the detection of the three‐dimensional organisation of VM and allow for the study of how these structures interact with the surrounding tumour microenvironment. Additionally, these imaging techniques can be used in conjunction with fluorescently tagged antibodies to visualise specific molecular markers associated with VM formation, providing insights into the molecular basis of VM [28]. In terms of morphological characteristics, the structure of VM usually presents a tubular shape, with no endothelial cells on its inner wall, but arranged with tumour cells. It can be determined by observing the structural morphology of tissue sections or under a microscope [29]. In addition to morphological features, the functionality of the structure is tested by observing the fluid flow inside the structure or injecting dyes to verify its vascular transport function. Recent efforts have focused on identifying specific biomarkers that could be used to detect and monitor VM in tumours. These biomarkers may include proteins involved in extracellular matrix (ECM) remodelling (e.g., MMPs), cell adhesion molecules, or specific signalling molecules that promote VM formation. The identification of reliable biomarkers could lead to the development of blood tests or imaging probes that enable the detection of VM in clinical settings, enhancing early diagnosis and monitoring treatment efficacy.

It should be noted that the structure of VM may have certain variability and complexity. Therefore, when determining whether it is VM, it is necessary to comprehensively consider multiple aspects of evidence and make a judgement based on the actual situation. However, it is currently believed that the PAS reaction is a necessary condition for determining VM.

### 2.2 Formation of VM

#### 2.2.1 Potential Mechanisms for VM Formation

The formation of tumour VM is an extremely complex process involving multiple potential mechanisms. Currently, most people believe that the formation of VM mainly involves the reshaping of cancer stem cells (CSCs), epithelial‐mesenchymal transition (EMT) and ECMs [30, 31]. Tumour stem cells are defined by the American Cancer Society as a type of cell with self‐renewal and multi‐directional differentiation potential in tumour cells [32]. CSCs may be the source of the vast majority of tumour cells present in malignant tumours, as well as the re‐acquisition of differentiated tumour cells through a process involving multiple mechanisms [33]. EMT is a reversible dedifferentiation process. Its characteristics include loss of cell connectivity and polarity, enhanced migration ability and acquisition of mesenchymal cell phenotype characteristics [34]. It usually occurs in epithelial cells and is seen in the occurrence of organs during normal embryonic development [33]. In general, epithelial cells do not possess the superpower to differentiate into different cells. But in some cases, epithelial cells also gain this super magic. Although EMT is a physiological reprogramming phenomenon of cells during development, cancer cells must not miss this magic. In recent years, a large number of studies have shown that EMT is closely related to the occurrence and development of tumours. Its activation can enhance the invasion and metastasis ability of tumour cells, thereby promoting their metastasis to distant organs [35].

**FIGURE 2:**
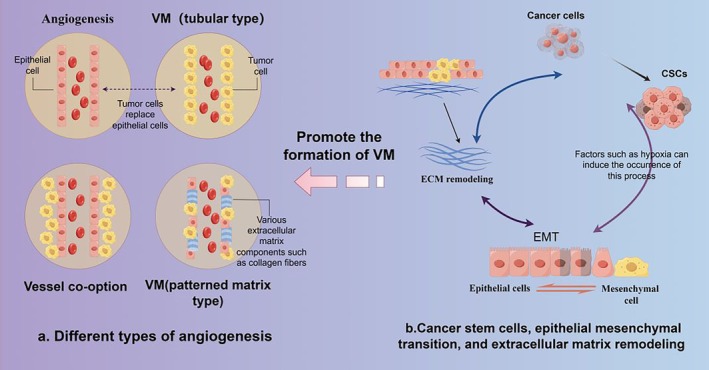
Types of angiogenesis and potential influencing mechanisms. (a) Different types of angiogenesis. (Angiogenesis: Endothelial cell recruitment forms blood vessels through which red blood cells can pass. Vessel co‐option: Tumour cells migrate along existing or newly induced blood vessels. Vasculogenic mimicry: VM is a tubular structure formed by tumour cells replacing endothelial cells. Tubular VM is composed of EC‐like tumour cells and covered by secreted glycoproteins, while the patterned stromal type is covered by PAS‐positive matrix). (b) Potential matrix for promoting VM formation. (Including the involvement of tumour stem cells, EMT and extracellular matrix remodelling.) (By Figdraw.)

During the formation of VM, CSCs promote the ability of tumour cells to form tubular structures by activating signalling pathways related to angiogenesis. Its plasticity is improved by inducing tumour cells to transition into the epithelial‐mesenchymal state, transforming into mesothelial cells with strong migration ability, thereby enhancing the formation of VM [36]. As cells undergo changes, the relevant transcription factors in the cells also change. Tumour cells express MMPs and other substances at high levels through a series of intracellular signalling pathways to degrade and reshape the surrounding ECM [37], promote tumour cell migration, and provide extension space and soil for the formation of the VM network. During the formation of VM, ECM not only provides support and structure but also regulates the behaviour of tumour cells through signal regulation and cell‐matrix interactions [38]. Meanwhile, the composition and mechanical properties of ECM can also affect the migration of cancer cells and the ability to form tubular structures, thereby affecting the formation and stability of VM. Some upregulated proteins arrange tumour cells through ductal adhesion, forming a ductal structure together with the reshaped extracellular matrix [39]. As this process gradually improves, these catheters penetrate and extend into the vascular network, beginning to transport red blood cells and nutrients to the interior of the tumour. Overall, CSCs, EMTs and ECM interact with each other during VM formation, jointly promoting the formation of vascular‐like structures in cancer cells and providing important support and conditions for tumour growth and metastasis (Figure 2).

#### 2.2.2 Signalling That Promotes VM Formation

##### 2.2.2.1 Matrix Metalloproteinases (MMPs)

MMPs are a large family, of which 26 members have been isolated and identified. MMPs are classified into 6 categories based on their substrates and fragment homology [40]. MMPs can degrade various protein components in the extracellular matrix (ECM) [41], disrupt the histological barrier of tumour cell invasion, and play a crucial role in tumour invasion and metastasis [42]. Among them, MMPs can degrade ECM, leading to the release of growth factors and cytokines embedded in ECM. These released factors may bind to receptors of surrounding tumour cells, thereby activating the PI3K/Akt signalling pathway and promoting VM formation [43]. Therefore, their role in tumour invasion and metastasis is increasingly valued, and they are considered the main proteolytic enzymes in this process. In gastric cancer, the formation of VM is positively correlated with levels of matrix metalloproteinase‐2, matrix metalloproteinase‐9, vascular endothelial growth factor (VEGF) and vascular endothelial growth factor receptor‐1 (VEGFR‐1) [44]. In addition, recent studies on the inhibition of VM by brucine in human cancer triple negative cell line mda‐mb‐231 suggest that brucine may inhibit VM by downregulating hepatocellular carcinoma A2, matrix metalloproteinase‐2 and metalloproteinase‐9 that produce erythropoietin [45].

##### 2.2.2.2 Vascular Endothelial Cadherin (VE‐Cadherin)

VE‐cadherin is a transmembrane adhesion protein specifically expressed on the surface of vascular endothelial cells [46], mediating adhesion between adjacent endothelial cells [47], and playing an important role in maintaining vascular integrity [48], regulating endothelial cell permeability, leukocyte exosmosis and intracellular signal transduction [49]. In addition to its adhesive properties, VE‐cadherin is also involved in regulating various intracellular processes, such as cell proliferation, and apoptosis, and regulating the function of vascular endothelial growth factor receptors [50]. Therefore, VE‐cadherin is necessary for angiogenesis during embryonic development and postnatal angiogenesis. In 2001, Hendrix et al. elucidated the biological significance of the expression and function of several endothelial‐related molecules in highly invasive and low invasive human skin melanoma cell lines. The downregulation of VE‐cadherin expression in invasive melanoma cells eliminates the ability of VM‐related network formation and directly tests the key hypothesis of VE‐cadherin in melanoma angiogenesis simulation VM [51]. The VE‐cadherin protein is highly expressed in high‐grade malignant melanoma cells, but not in low‐level malignant melanoma cells [52]. Inhibiting the expression of VE‐cadherin using thiosulfate‐modified oligonucleotides can block the formation of vascular mimicry in high‐grade malignant melanoma [51].

##### 2.2.2.3 Vascular Endothelial Growth Factor

**FIGURE 3:**
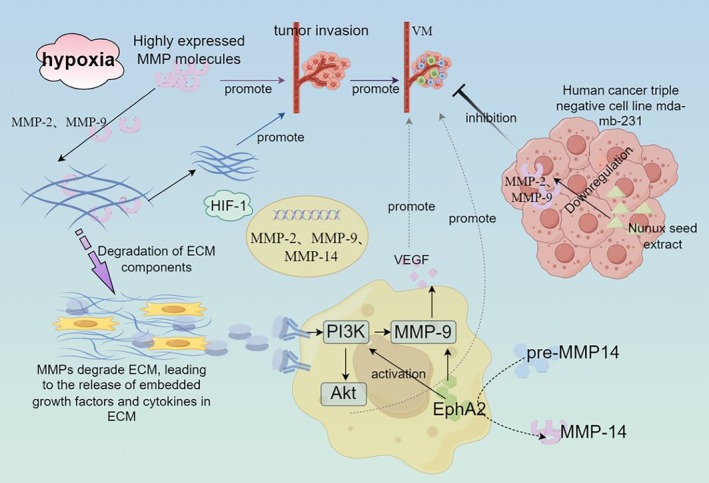
Signal promoting VM formation. Under hypoxic conditions, MMPs‐2 and MMPs‐9 are highly expressed. MMPs can degrade various protein components in the ECM, leading to the release of growth factors and cytokines embedded in the ECM. These released factors may bind to receptors of surrounding tumour cells, thereby activating the PI3K/Akt signalling pathway and promoting VM formation. In addition, EphA2 can activate PI3K and promote the generation of MMP‐9 and VEGF. And under the action of EphA2, pre‐MMP14 can be converted to MMP‐14. In addition, in the human cancer triple negative cell line mda‐mb‐231, brucine may inhibit VM by downregulating matrix metalloproteinase‐2 and metalloproteinase‐9. (By Figdraw.)

VEGF is a highly specific vascular endothelial cell growth factor that promotes increased vascular permeability, extracellular matrix degeneration, endothelial cell migration, proliferation and angiogenesis [53]. VEGF is a big family. VEGF‐A can promote neovascularization and increase vascular permeability [54]. VEGF‐B plays a role in non‐neovascularized tumours [55], VEGF‐C and VEGF‐D play a role in the formation of new blood vessels and new lymphatic vessels in cancer tissue [56], and VEGF‐E is also a potential neovascularization factor [57]. PGF promotes neovascularization and increases vascular permeability. Its related receptors VEGFR‐1 and VEGFR‐2 are mainly distributed on the surface of tumour vascular endothelium, regulating the generation of tumour blood vessels [58]; VEGFR‐3 is mainly distributed on the surface of lymphatic endothelium and regulates the generation of tumour lymphatic vessels [59]. For example, low expression of VEGF‐C can inhibit the proliferation, VM and EMT of nasopharyngeal carcinoma cells [60]. Glioblastoma (GBM) cells promote angiogenesis mimicry and tumour development through activation of VEGFR‐2 (Flk‐1) (Figure 3 and Table 2). The vascular channels of VM in GBM are composed of tumour cells strongly expressing Flk‐1 [61].

**TABLE 2:** Signal molecules that affect the formation of VM.

Molecular signal	Target	Mechanism	Effect of VM formation	Associated cancer types	Reference
Matrix metalloproteinases (MMPs)	PI3K/Akt	Degrades extracellular matrix components, creating vascular‐like channels, promoting VM formation	Promotion	Liver cancer, gastric cancer, breast cancer, etc	[62]
VE‐cadherin	β‐catenin/TCF‐4	VE‐Cadherin modulates β‐catenin/TCF‐4 to enhance VM	Promotion	Melanoma	[63]
HMGA2	Twist1 and VE‐cadherin	HMGA2 induces the expression of Twist1 and VE‐cadherin to enhance VM	Promotion	Gastric cancer	[64]
CD36	—	CD36 supports VM formation by human melanoma cells as well as adhesion to, and invasion through, a cancer derived extracellular matrix substrate	Promotion	Melanoma	[65]
microRNA‐29b	MMP‐2, MMP‐9 and VEGF	MicroRNA‐29b reduced the expression of MMP‐2, MMP‐9 and VEGF, as well as inhibiting EMT	Inhibition	Colorectal cancer	[66]
Vitamin D binding protein (VDBP)	Twist1	VDBP hindered the binding of Twist1 on the promoter of VE‐cadherin by interacting with its helix–loop–helix DNA binding domain, ultimately leading to the inhibition of VM	Inhibition	Hepatocellular carcinoma	[67]

## 3 VM: An Accomplice to Tumour Progression

Tumours are a serious threat to human health, and their malignancy is mainly manifested in invasive growth, distant metastasis and resistance to treatment [68]. In the complex network of tumour progression, VM is a key factor that widely participates in and promotes tumour development. Below, we will comprehensively explore three aspects: promoting tumour invasion and metastasis through VM, improving tumour resistance and to some extent determining tumour prognosis.

### 3.1 VM Promotes Tumour Invasion and Metastasis

Long‐distance metastasis of tumours is one of their lethal characteristics. VM involves a tubular structure with a basement membrane that resembles and communicates with blood vessels but functions independently of blood vessels to nourish tumour cells, promote tumour progression, invasion, and metastasis, and reduce 5‐year survival rates [69]. The generation of VM not only provides a material basis for the rapid growth of tumours but also provides a pathway for the invasion of tumour cells. In addition, VM can provide a pathway for tumours to evade immune system attacks [70]. Due to these channels not being true blood vessels, the immune system may not be able to effectively recognise and attack them, making it easier for tumour cells to spread in the body. Immune cells such as lymphocytes and macrophages are usually located in the interstitial tissue around tumours and interact with tumour cells and their surrounding blood vessels [71]. These immune cells play an important role in tumour immune surveillance and anti‐tumour response, but they do not directly enter the VM pipeline composed of tumour cells. VM can also enhance the activity of MMPs by altering cell adhesion, making it easier for tumour cells to penetrate the vascular wall, enter the circulatory system and spread to other parts of the body through blood flow, forming distant secondary lesions [63]. In gallbladder cancer, STAT3 expression is positively correlated with VM. The likelihood of early postoperative recurrence of VM‐positive gallbladder cancer is higher, and the incidence of VM in the group with poor histological grading is significantly higher than that in the group with good histological grading [72].

### 3.2 VM Enhances Tumour Drug Resistance

One of the main methods for treating tumours is to use anticancer drugs, but the resistance of tumour cells to these drugs often becomes a challenge in treatment [73]. VM improves the tumour microenvironment and enhances the drug resistance of tumour cells by increasing the supply of nutrients and oxygen within the tumour. Additionally, the presence of new blood vessels also increases the number of deep tumour cells that are difficult for drugs to reach, thereby reducing the therapeutic effect [74]. Compared with traditional angiogenesis, VM is a pipeline or channel directly formed by tumour cells and does not involve endothelial cells. Therefore, the resistance mechanism of VM may be more related to the characteristics and functions of tumour cells. In addition, due to differences in drug targets involved, VM and conventional angiogenesis may develop different resistance mechanisms against angiogenic drugs. For example, due to different sources, VM may produce different responses to drugs that inhibit endothelial cell proliferation or function.

### 3.3 VM Determines Tumour Prognosis to a Certain Extent

Rich angiogenesis not only provides survival conditions for tumours, but is also closely related to the malignancy, growth rate and response to treatment of tumours. Therefore, VM can serve as an important indicator for evaluating tumour prognosis to a certain extent. Some studies have shown a correlation between VM and patient survival in gastric cancer patients. VM positivity indicates poor prognosis in gastric cancer patients, which can lead to higher TNM clinical staging (stage III or IV) and significant heterogeneity. What's more, there is a significant correlation between VM positivity and lymph node metastasis in GC patients. VM positivity can induce poor pathological differentiation and is significantly associated with blood metastasis in gastric cancer patients [75]. Some studies have found that the presence of VM in breast cancer patients is closely related to the depth of tumour invasion, metastasis and patient prognosis. VM‐positive breast cancer is usually associated with a higher recurrence rate and poor survival [76]. In malignant tumours such as gliomas, VM positivity is often closely related to vascular invasion, local recurrence and poorer patient survival [77].

VM is closely related to the prognosis of various types of cancer and is usually associated with tumour invasiveness, metastasis tendency and patient survival rate. Therefore, VM can serve as an important prognostic indicator to evaluate the prognosis of tumour patients and guide the development of clinical treatment strategies.

## 4 The Impact of the Immune System on VM

In VM, tumour cells exhibit characteristics similar to endothelial cells, forming vascular structures composed of tumour cells rather than normal endothelial cells. The immune system usually monitors and clears abnormal cells, including tumour cells. Tumour cells can also evade immune system attacks through various mechanisms. Therefore, in this case, immune cells may also be involved in regulating the process of VM.

### 4.1 The Role of Immune Cells in VM Regulation

In VM, tumour cells or other tumour‐related cells can form endothelial‐like structures, providing oxygen and nutrients to the tumour [78] and may also provide a pathway for escaping immune surveillance [79]. The role of lymphocytes in simulating VM angiogenesis has not been fully elucidated, but some studies have pointed out that the impact of lymphocytes on VM is complex and diverse, and can be explained based on their type and state. CD4+T cells play an important role in regulating immune responses, and their activity can affect the angiogenesis process in the tumour microenvironment. Activated CD4+T cells may release various cytokines and signalling molecules, such as IL‐2 and IFN‐γ. Wait to affect the growth and function of tumour cells, thereby inhibiting the formation of VM [80]. CD8+T cells and NK cells can kill tumour cells [81]. Wait to inhibit tumour growth, which may inhibit the formation of VM. Treg cells play an important role in regulating immune tolerance and suppressing immune responses. High levels of Treg cells may promote tumour growth by inhibiting the activity of CD4+ and CD8+T cells [82], which may contribute to the formation of VM. Overall, the impact of lymphocytes on VM depends on their type, quantity, and activity status in the tumour microenvironment. Active CD4+ and CD8+T cells, as well as NK cells, may inhibit the formation of VM, while regulatory T cells may promote the development of VM. Therefore, in tumour treatment, regulating the immune function of lymphocytes may be one of the important strategies that affect VM and tumour progression.

**FIGURE 4:**
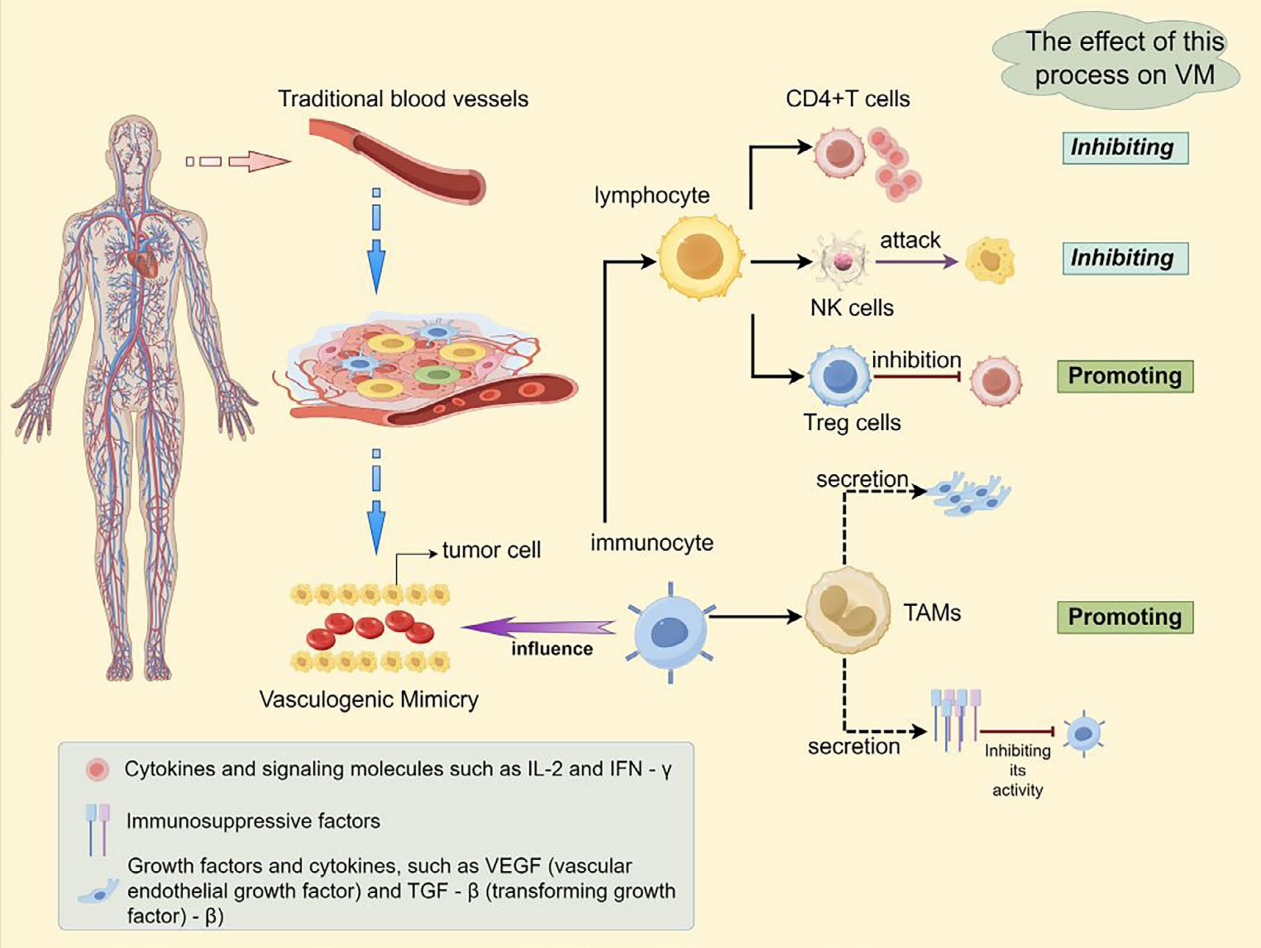
The role of immune cells in VM regulation. The impact of lymphocytes on VM is complex and diverse. (Activated CD4 + T cells can release various cytokines and signalling molecules, such as IL‐2 and IFN‐γ. They affect the growth and function of tumour cells, thereby inhibiting VM formation. NK cells can kill tumour cells and inhibit VM formation. High levels of Treg cells can promote tumour growth by inhibiting the activity of CD4+ and CD8+T cells, which contributes to the formation of VM.) TAMs also affect the formation of VMs. (It can secrete various growth factors and cytokines, such as VEGF, TGF‐β, FGF, etc., which can promote tumour angiogenesis and vascular remodelling, thereby promoting the occurrence of VM. On the other hand, TAMs can secrete immunosuppressive factors such as IL‐10 and TGF‐β, which inhibit the activity of immune cells and provide favourable conditions for the formation of VM.) (By Figdraw.)

In addition, tumour‐associated macrophages (TAMs) play an important role in VM, which mainly includes the following aspects. TAMs can secrete various growth factors and cytokines, such as VEGF and transforming growth factor‐β (TGF‐β), fibroblast growth factor (FGF) and other factors can promote angiogenesis and vascular remodelling within tumours [83], thereby promoting the occurrence of VM. They affect the growth, migration and pipeline formation of tumour cells by secreting these factors, thereby supporting the VM process. TAMs can remodel the tumour stroma and microenvironment by secreting various proteases, such as metalloproteinases (such as MMPs) and proteasome proteases, which can degrade protein components in the extracellular matrix, such as collagen and fibronectin, thereby disrupting the integrity of the extracellular matrix. The changes in the surrounding stromal environment of tumours support the formation of VM [83]. The remodelling of this matrix helps tumour cells reorganise in three‐dimensional space, forming a pipeline structure. TAMs can also induce EMT in tumour cells. By secreting factors such as TGF‐β and IL‐6, TAMs promote EMT, endowing tumour cells with enhanced migratory and invasive abilities. The immunomodulatory effect of TAMs may also affect VM. TAMs can secrete immunosuppressive factors such as IL‐10 and TGF‐β [84, 85]. These factors can inhibit the activity of immune cells and reduce the immune system's ability to monitor and attack tumours. Through this approach, TAMs can reduce the infiltration and activity of immune cells, providing favourable conditions for the formation of VM. In addition, TAMs may affect their function and activity through interactions with other immune cells, such as T cells and natural killer cells. This may result in immune cells being unable to effectively clear tumour cells, providing favourable conditions for the formation of VM. Overall, TAMs participate in the regulation of VM through various means, thereby affecting the growth and development of tumours. Understanding the role of TAMs in VM helps to reveal the diverse mechanisms of angiogenesis in the tumour microenvironment, providing a theoretical basis for developing relevant therapeutic strategies (Figure 4).

### 4.2 VM and Immune Checkpoints

Recent studies have unveiled a potential link between VM and immune checkpoints, particularly programmed death‐ligand 1 (PD‐L1). PD‐L1 is a key molecule involved in immune evasion, frequently overexpressed in tumour cells to suppress T‐cell‐mediated immune responses [86]. Tumour cells exhibiting VM structures often demonstrate increased PD‐L1 expression, suggesting a synergistic mechanism by which these cells escape immune surveillance.

The relationship between VM and PD‐L1 may involve multiple pathways. For instance, the hypoxic tumour microenvironment, a known inducer of VM, also upregulates PD‐L1 expression through hypoxia‐inducible factor‐1α (HIF‐1α) signalling [87]. Hypoxia not only promotes VM formation by enhancing tumour cell plasticity but also reinforces immune evasion by inducing immune checkpoint molecules [88]. Consequently, VM‐associated tumour cells may exploit PD‐L1 expression to evade immune destruction, further promoting tumour progression and metastasis. Moreover, studies have observed a correlation between high VM density and reduced infiltration of cytotoxic T cells (CTLs), such as CD8+ T cells. This reduced immune infiltration is frequently accompanied by elevated PD‐L1 levels, which inhibit T‐cell activation and function. The VM structures, serving as non‐traditional vascular channels, may further protect tumour cells from immune cell attacks by providing a physical barrier that limits CTL access.

In addition to PD‐L1, other immune checkpoint molecules, such as CTLA‐4 and LAG‐3, may also interact with VM‐related mechanisms. For example, tumour cells forming VM may express ligands that engage these checkpoints, further dampening the immune response [89]. These findings underscore the complex interplay between VM, immune checkpoints and immune cell dynamics in the tumour microenvironment.

### 4.3 VM and Immune Infiltration

Immune infiltration refers to the process in which immune cells (such as T cells, B cells, macrophages, etc.) pass through blood vessels and enter tissues to combat abnormal conditions such as infections and tumours, which are related to the normal function and immune response of the immune system. More and more evidence suggests that immune cell infiltration in the tumour microenvironment is closely related to tumour development and treatment response. For example, infiltration of T cells is considered a good prognostic factor as they can recognise and kill tumour cells [90].

VM is a non‐classical pathway for tumour angiogenesis. Some studies have found that VM is closely related to the immune infiltration status of tumours, especially to the infiltration status of T cells. Specifically, some studies suggest that in tumours with high VM, there may be less T cell infiltration, while in tumours with low VM, T cell infiltration may be more abundant [91]. This association may imply that VM has an impact on the immune microenvironment of tumours, thereby affecting their response to immunotherapy. Some studies are exploring how to utilise this association to develop more effective tumour immunotherapy strategies. For example, in gastric cancer, both heatmaps and box plots of 29 immune cells show a correlation between VM index and immune response. The experiment detected the relationship between immune cells and checkpoints with VM index, and the results showed that the expression of immune checkpoints such as CD28, CD86, BTLA, CD40LG, CD4 and CD8A was positively correlated with VM index. This suggests that VM may promote the onset and metastasis of GC by regulating immune cells and immune monitoring. In addition, the experiment also found that the upregulation of VM was accompanied by higher immune infiltration indicators, indicating the necessity of tumour stem cells and immune infiltration for VM to regulate GC cells [9]. In addition, compared with VM negative tumours, VM positive tumours have reduced anti‐tumour immune cell activity, decreased infiltration of CD8+T cells and natural killer (NK) cells, and weakened the immune system's ability to combat VM positive tumours. CD8+T cells in TME typically exhibit a state of functional exhaustion, characterised by high expression of immune checkpoints such as PD‐1 and CTLA‐4. From a therapeutic perspective, the immunosuppressive environment in VM positive tumours reduces the efficacy of immune checkpoint inhibitors such as anti‐PD‐1/PD‐L1 and anti‐CTLA‐4 therapy. The high levels of Tregs and MDSCs weaken the activation of effector T cells, limiting the therapeutic effect [89]. Therefore, studying the relationship between VM and immune infiltration is of great significance for us to understand the immune escape mechanism of tumours and develop new immunotherapy strategies.

## 5 Clinical Significance of the Interaction Between VM and the Immune System

### 5.1 Clinical Application of VM

VM plays an important role in the diagnosis, prognosis evaluation and treatment effect monitoring of tumours. First, VM can serve as an indicator for tumour diagnosis and prognostic evaluation [92]. The presence of VM can help distinguish between malignant tumours and benign lesions [93]. Through histological observation and imaging techniques, the VM structure in tumour tissue can be detected [93], thereby assisting in diagnosing tumours. For example, VM typically manifests as a lack of endothelial cell channels or channel networks, which are formed by tumour cells or other cell types. These channels can be observed through optical microscopy, electron microscopy, magnetic resonance imaging (MRI), etc. In terms of diagnosis, the best imaging techniques may depend on the type, location and individual condition of the tumour. For example, optical microscopy may be a commonly used and effective method for observing VM structure on tissue sections. MRI and CT scans may be more suitable for observing larger VM structures in vivo. The difference between benign and malignant VM structures may include the size, morphology and activity of surrounding cells of VM channels. A benign VM is usually a physiological structure, while a malignant VM is usually associated with tumour invasiveness and prognosis. However, accuracy and the feasibility of clinical application remain challenges. Although some studies suggest that VM structures may be associated with tumour diagnosis and prognosis, the results of these studies require more validation and clinical practice to confirm their accuracy and practicality. At present, the clinical application of VM structure for tumour diagnosis is still in the research and development stage, and it may take some time before it is put into clinical use. In addition, many studies have shown that VM is closely related to tumour invasiveness and prognosis [43]. The degree of presence of VM in tumour tissue is related to the patient's survival period and the tendency of tumour metastasis [94]. The presence of VM often indicates that the tumour is more aggressive and the patient's prognosis is worse [95]. Gliomas [96], breast cancer [96] and other types of tumours have manifestations. Secondly, VM can serve as a target for tumour therapy [97]. Due to the important role of VM in the occurrence and development of tumours, inhibiting the formation or signalling pathways of VM has become an important direction in tumour treatment. Researchers are attempting to develop a drug that can specifically inhibit VM formation, targeting VM inhibition to improve the effectiveness of tumour treatment [18]. At present, research on inhibitory drugs for VM is still in its early stages, but there are already some potential candidate drugs and research directions emerging. Mainly including anti‐angiogenic drugs and targeted drugs targeting specific signalling pathways. These drugs mainly target the relevant signalling pathways or specific molecules of tumour cells, such as the PI3K/Akt signalling pathway, VEGF signalling pathway, etc., to interfere with the ability of tumour cells to form VM. At present, research on inhibitory drugs for VM is still in the laboratory and animal model stages. Although some candidate drugs have shown certain inhibitory effects on VM, further validation and clinical trials are needed for their clinical application. Finally, VM is also an indicator for monitoring the effectiveness of tumour treatment. The existence and development of VM are closely related to the growth and metastasis of tumours. Therefore, monitoring changes in VM can serve as one of the indicators for evaluating the effectiveness of tumour treatment. For example, observing changes in the morphology and quantity of VM within tumours through imaging techniques can help evaluate the effectiveness of treatment [98]. Observing the morphological and quantitative changes of VM structures within tumours through imaging techniques can help evaluate the effectiveness of treatment. Here are some commonly used imaging techniques and methods. MRI can provide high‐resolution tumour images, allowing observation of the vascular structure inside the tumour. Through MRI scanning, the morphology and distribution of VM structure can be observed, as well as changes with treatment progress. Ultrasound can provide real‐time tumour images, especially suitable for observing blood flow dynamics. Ultrasound can evaluate the morphology and quantity of blood vessels inside tumours, and monitor changes in VM. Optical microscopy can be used to observe the microvascular structure in tissue slices. The quantity and morphology of VM can be quantitatively evaluated through microscopic observation, and samples at different treatment time points can be compared. These technologies also have different cost‐effectiveness impacts. For example, optical microscopy is cost‐effective for histological analysis, but MRI and CT scans, while offering in vivo visualisation of larger VM structures, involve higher costs that may limit accessibility in resource‐limited settings. Traditional histological techniques, such as PAS staining, are relatively inexpensive and widely used in pathology laboratories. Therefore, selecting techniques should balance diagnostic accuracy and economic feasibility, tailored to the type and stage of tumours. When conducting imaging observations, it is necessary to pay attention to regular monitoring, quantitative analysis and correlation with clinical data to guide the adjustment and optimization of treatment plans. In addition, based on changes in VM, doctors can predict the tumour's response to specific treatment methods and adjust treatment plans accordingly to achieve better treatment outcomes.

In summary, VM is of great significance in tumour diagnosis, prognosis evaluation, treatment target selection and treatment effect monitoring. With in‐depth research on the mechanism of VM, it is believed that VM will play an increasingly important role in clinical practice, providing new ideas and methods for tumour treatment (Table 3).

**TABLE 3:** Theoretical imaging techniques and methods that can be used to observe VM.

Technique	Applicable cancer types	Principle	Conditions	Advantages	Disadvantages	Ref.
X‐ray angiography	Brain, liver, colorectal, etc	Uses contrast agents injected into blood vessels, and utilises x‐rays to visualise vascular structures	Requires contrast agents, x‐ray sensitive, may require anaesthesia	Accurate depiction of vascular morphology and blood flow	Radiation exposure, potential risks associated with contrast agents	[99]
CT Angiography	Brain, liver, colorectal, etc	Generates 3D images of vascular structures through x‐ray scanning and computer processing	Limited by patient's weight, requires contrast agents	Fast imaging, high‐resolution vascular images	Higher radiation dose, potential radiation risks	
Magnetic resonance Angiography (MRA/MRI)	Brain, breast, liver, etc	Utilises MRI technology, does not require radioactive contrast agents, visualises vascular structures and blood flow	Not suitable for patients with implanted devices like pacemakers	No radiation, no need for contrast agents, provides high‐contrast vascular images	MRI may not be suitable for certain patients, for example, those with metal implants or pacemakers	[100, 101]
Ultrasound imaging	Breast, kidney, prostate, etc	Utilises ultrasound waves to observe vascular structures and blood flow	No need for radiation or contrast agents, and no radiation risk	Safe, non‐invasive, real‐time observation of blood flow	Relatively lower resolution, susceptible to interference from body fat and gas	[102]
Optical coherence tomography (OCT)	Breast, skin, etc	Observe microvascular structures using OCT technology	Suitable for superficial tissue examination, such as skin cancer	High‐resolution, real‐time observation of microvascular morphology	Invasive, limited to superficial tissue observation	[103]
Nuclear imaging	Bone, thyroid, lymphoma, etc	Utilises radioisotope‐labelled molecules to image vascular structures and function	Dependent on radioisotope half‐life and biological distribution	Provides biological information, high sensitivity for specific cancers	Radiation exposure, longer imaging times, relatively lower resolution	[104]
Microscopy	All cancer types	Uses optical magnification to observe microscopic structures in tissue samples, including vascular structures	Requires tissue samples and histological processing	Provides high‐resolution images of microstructure, direct observation of vascular morphology and density	Invasive, not suitable for real‐time observation	[105]

### 5.2 Treatment Strategies for VM and Immune System

#### 5.2.1 Anti‐Angiogenic Therapy and Its Impact on VM

Anti‐angiogenic therapy is a therapeutic strategy aimed at inhibiting tumour growth and metastasis, targeting tumour angiogenesis. These therapies mainly include anti‐angiogenic factors and anti‐angiogenic agents [106], which affect tumour angiogenesis through different mechanisms. Anti‐angiogenic factor therapy prevents the formation and growth of tumour blood vessels by inhibiting the production or action of angiogenic factors required for tumour growth [107]. Among them, the most widely studied and applied anti‐angiogenic factor is VEGF and its receptor. Anti‐angiogenic factor therapy can be administered intravenously or orally, such as bevacizumab. And anti‐angiogenic therapy directly interferes with the generation and stability of tumour blood vessels to achieve the goal of inhibiting tumour growth [108]. They can block the proliferation of endothelial cells, promote vascular apoptosis, or destroy the formed blood vessels [109]. Typical anti‐angiogenic agents include Erlotinib and Toceranib, which can inhibit tumour angiogenesis and growth through various mechanisms.

Research has shown that anti‐angiogenic therapy can significantly inhibit the formation of VM in tumours. This is because the formation of VM structure is also regulated by angiogenic factors [110], and inhibiting the activity of angiogenic factors can interfere with the formation and development of VM structure. Thus, reducing the invasiveness and metastatic tendency of tumours, and improving the prognosis of patients. Anti‐angiogenic therapy is usually used in combination with other treatment methods such as chemotherapy, radiotherapy, etc., which can synergistically enhance the control effect on tumours [111]. The combination of these treatments can affect tumour angiogenesis and VM formation through multiple pathways. For example, treatment methods such as chemotherapy and radiation therapy can directly affect the proliferation and survival ability of tumour cells, thereby reducing tumour cell metastasis and slowing down disease progression. The combined use of anti‐angiogenic therapy can reduce tumour resistance and enhance the effectiveness of these treatment methods.

#### 5.2.2 Immunotherapy and Its Potential in Regulating VM

Immunotherapy is a novel tumour treatment strategy that attacks and kills tumour cells by regulating the patient's immune system [112]. The main types of immunotherapy include immune checkpoint inhibitors, CAR‐T cell therapy, tumour vaccines, etc. [113]. These therapies have shown encouraging effects in various cancer treatments.

The potential of immunotherapy in regulating VM lies in the following aspects. Firstly, immunotherapy can affect the formation of VM by regulating the immune response in the tumour microenvironment [114]. Some studies have shown that immunotherapy can inhibit the transformation of tumour cells into vascular‐like structures and reduce the formation of VM. Especially, by activating immune cells such as T cells and natural killer cells, they can be prompted to eliminate tumour cells that have been transformed into tubular structures within the tumour [115]. In tumour tissue, if there are tubular structures, these structures may be considered abnormal by the immune system and activate the immune response of T and NK cells. This reaction may be due to certain specific antigens or markers on the surface of the VM structure, or because the VM structure disrupts the normal structure and function of tumour tissue. However, it is currently unclear how T cells and NK cells preferentially recognise tumour cells with tubular structures, and further research is needed to elucidate their mechanisms. In addition, immunotherapy can enhance the patient's immune response, thereby reducing the invasiveness and metastatic tendency of tumours, and improving the patient's prognosis [116]. Another promising approach is combining immune checkpoint inhibitors (such as anti‐PD‐1 or anti‐PD‐L1 antibodies) with VM‐targeted therapies. For instance, in preclinical cancer models, the combination of anti‐PD‐1 therapy with anti‐angiogenic agents targeting the VEGF pathway can reduce the formation of VM and enhance anti‐tumour immunity [117]. This combined therapy disrupts immune evasion mechanisms within the tumour microenvironment while increasing immune cell infiltration into tumours, thereby improving therapeutic efficacy. In trials involving cancers like non‐small cell lung cancer (NSCLC), combining anti‐VEGF therapy with anti‐PD‐L1 inhibitors showed that patients with high VM density experienced a significant reduction in VM formation and improved survival rates, providing a foundation for further exploration of such therapies [118]. Beyond PD‐1/PD‐L1 inhibitors, VM‐targeted therapies have also been combined with cytokine‐based treatments (e.g., IL‐2 or interferon). For example, in melanoma models, IL‐2 combined with anti‐angiogenic therapy inhibited VM formation, reducing metastasis in the tumour microenvironment and enhancing immune activity [119]. The presence of VM is often closely related to the invasiveness and prognosis of tumours, therefore, the effectiveness of immunotherapy may affect the formation and development of VM in tumours.

Although immunotherapy has made significant progress in tumour treatment, it still faces some challenges, such as immune escape, immune tolerance, etc [120]. Therefore, further research and exploration are needed to explore the potential of immunotherapy in regulating VM. Future research may focus on the combined application of immunotherapy and other treatment methods, as well as the mechanism of immunotherapy in regulating the tumour microenvironment, to better utilise immunotherapy to regulate VM and improve the effectiveness of tumour treatment.

### 5.3 Challenges and Limitations of Targeting VM and Immune Response in Cancer Treatment

Targeted VM and immune response as cancer treatment strategies have gradually entered people's field of vision in recent years, becoming a key research direction. However, their related research still has many potential challenges and limitations.

Firstly, the impact of the complex microenvironment of tumours. The tumour microenvironment is very complex, including immunosuppressive cells, immunosuppressive factors, etc. [121], which may interfere with the effectiveness of immunotherapy. In this microenvironment, immune cells of immunotherapy may not be able to effectively recognise and attack tumour cells [122]. Secondly, tumour cells can evade recognition and attack by the immune system in various ways, such as reducing antigen expression and enhancing the secretion of immunosuppressive factors. In addition, although we have a preliminary understanding of some mechanisms of VM, our understanding of its detailed molecular mechanisms and regulatory pathways is still limited. Lack of in‐depth understanding of VM regulation mechanisms may lead to treatment uncertainty, making it difficult to accurately treat and prevent VM. In addition, current technologies for detecting and diagnosing VM still face some challenges, including insufficient imaging resolution and insufficient specific markers. Therefore, it is necessary to further develop more accurate and reliable detection methods and equipment. Although some small‐scale clinical studies have suggested that VM may be related to tumour prognosis and treatment response, there is currently a lack of large‐scale, multicenter clinical data to verify the effectiveness and reliability of its clinical application. It is worth mentioning that drug resistance, individual differences and inconsistent clinical trial results in tumour treatment have increased the difficulty of research, and the combination of the two treatment methods greatly improves the toxicity of drugs to cells.

All in all, although targeting angiogenesis mimicry and immune response as a new strategy for cancer treatment has great potential, it still faces many challenges and limitations. Future research needs to delve deeper into these challenges and limitations to find more effective treatment strategies. Develop more accurate and effective diagnostic and treatment methods to improve the treatment effectiveness and survival rate of cancer patients.

## 6 Conclusion and Further Prospects

Cancer is a highly complex disease, and its treatment and prognosis are influenced by multiple factors. VM and immune response are two important aspects of cancer treatment and prognosis. VM is a nonendothelial cell‐dependent vascular morphology that provides oxygen and nutrients to tumours by stimulating angiogenesis. The mechanism of VM formation is not fully understood, but it may be related to factors such as tumour stem cells, EMT and extracellular matrix remodelling. VM not only promotes tumour invasion and metastasis but is also closely related to tumour prognosis. The immune system plays an important regulatory role in the generation of VM. Immune cells, such as TAMs and lymphocytes, as well as other cell types, such as adipocytes and fibroblasts, can affect the formation and development of VM. For example, CD4+T cells and NK cells can inhibit the generation of VM. TAMs can accelerate the formation of VMs. In addition, immunotherapy activates the immune system to recognise and eliminate tumour cells, while also potentially affecting the formation and stability of VM.

The integration of VM and immune response is of great significance for the treatment and prognosis of cancer. Firstly, by gaining a deeper understanding of the mutual interference mechanism between VM and the immune system, further research can be conducted on related pathway targets. Secondly, related ligand receptors can serve as prognostic markers or therapeutic targets, providing direction for further research and guidance for developing more effective treatment strategies. Secondly, the use of anti‐angiogenic therapy and immunotherapy can simultaneously affect tumour angiogenesis and immune response, thereby better controlling tumour growth and metastasis. However, targeting VM and immune response still face some challenges and limitations in cancer treatment, requiring further research and exploration. Firstly, one of the primary challenges in translating VM targeted therapy into clinical practice is drug specificity. The characteristic of VM is the formation of endothelial like structures composed of tumour cells, rather than typical endothelial cells. Therefore, targeting VM requires the development of highly specific drugs to distinguish between tumour cells that form VM and normal endothelial cells involved in conventional angiogenesis. Although some VM targeted drugs, such as those targeting extracellular matrix or MMPs, have shown promise in preclinical models, their selective inhibition remains a major obstacle. Another major challenge in developing effective VM targeted therapies is the possibility of drug resistance. Tumours typically adapt to treatment by utilising alternative blood supply mechanisms, including activating compensatory angiogenesis pathways or recombinant extracellular matrix. In addition, VM itself has high plasticity, which means that tumours may alter their VM structure to respond to treatment, rendering the initial treatment ineffective. This adaptability emphasises the necessity of combination therapies targeting VM and other angiogenesis related pathways. The resistance mechanism also extends to the immune response. Tumours that rely on VM to provide nutrition and oxygen supply may exhibit immune evasion strategies, such as upregulating immune checkpoints such as PD‐L1, which can reduce the effectiveness of immunotherapy. Understanding the interaction between VM and immune evasion is crucial for overcoming these resistance mechanisms and improving the efficacy of combination therapy. Given the heterogeneity of different cancer types and the varying expression of VM, patient stratification is crucial for optimising the outcomes of VM targeted therapy. Not all tumours exhibit VM, and even in those tumours that exhibit VM, there are significant differences in the degree and nature of VM. Stratifying patients based on the presence, density and type of VM can help determine the individuals most likely to benefit from VM targeted therapy. Predicting the formation of VM and its biomarkers for treatment response is crucial for personalised treatment strategies.

Based on the study of the interaction between VM and the immune system, we may be able to predict biomarkers and immune responses in cancer patients, develop targeted therapies for VM disruption and enhancement of immune responses, and explore the application prospects of VM in personalised healthcare. Firstly, there is research on new methods. New methods include high‐throughput imaging techniques, single‐cell analysis, bioinformatics tools, etc., which can more comprehensively reveal the interaction between VM and the immune system. Combining knowledge from multiple disciplines such as bioinformatics, systems biology and biomedical engineering can help to gain a deeper understanding of the interaction between VM and the immune system. In addition, by identifying biomarkers related to VM and the immune system, early detection of changes in cancer patients can be achieved, and personalised treatment guidance can be provided. By combining genomics, proteomics and metabolomics analysis, more accurate biomarkers can be discovered, providing a more reliable basis for personalised healthcare. In addition, targeted therapy for VM can be developed to weaken the tumour's blood supply and reduce the likelihood of tumour growth and metastasis. Design immune modulators and immune checkpoint inhibitors to enhance the immune system's ability to recognise and clear tumours. Finally, we also hope to have good application prospects in personalised healthcare. For example, based on the characteristics of individual VM and immune systems, precision medicine can be carried out to provide more effective treatment plans for patients. By monitoring changes in VM and immune system, predict patient prognosis and efficacy, and adjust treatment plans promptly. Combining imaging and biomarker monitoring techniques, track changes in VM and immune system during treatment, and evaluate treatment efficacy and potential side effects.

Overall, through in‐depth research on the interaction between VM and the immune system, we may be able to predict and intervene in the development process of tumours, provide new ideas and methods for cancer treatment, and ultimately achieve the goal of personalised healthcare.

## Author Contributions

Z.Q.L., Y.Q.R. and T.P. provided direction and guidance throughout the preparation of this manuscript. M.K., Y.Q.R., S.T.L. and L.G. wrote and edited the manuscript. S.T.L., T.P. and L.G. reviewed and made significant revisions to the manuscript. X.W.H., and Z.Q.L. revised and edited the manuscript. Y.Y.Z., Y.H.B., J.H.D., P.L., Q.C., H.X., S.Y.W., A.N.Z. and S.T.L. collected and prepared the related papers. All authors read and approved the final manuscript.

## Conflicts of Interest

The authors declare no conflicts of interest.

## Data Availability

Data sharing not applicable to this article as no datasets were generated or analysed during the current study.
